# Collagen’s primary structure determines collagen:HSP47 complex stoichiometry

**DOI:** 10.1016/j.jbc.2021.101169

**Published:** 2021-09-04

**Authors:** Elena T. Abraham, Sinan Oecal, Matthias Mörgelin, Philipp W.N. Schmid, Johannes Buchner, Ulrich Baumann, Jan M. Gebauer

**Affiliations:** 1 Faculty of Mathematics and Natural Sciences, Institute of Biochemistry, University of Cologne, Cologne, Germany; 2 Division of Infection Medicine, Department of Clinical Sciences, Lund University, Lund, Sweden; 3 Colzyx AB, Lund, Sweden; 4 Department of Chemistry, Center for Integrated Protein Science, Technische Universität München, Garching, Germany

**Keywords:** collagen, extracellular matrix proteins, protein–protein interaction, crystal structure, stoichiometry, AUC, analytical ultracentrifugation, CMP, collagen model peptide, COL1A1, collagen type I alpha 1 chain, COL1A2, collagen type I alpha 2 chain, COL2A1, collagen type II alpha 1 chain, COL5A2, collagen type V alpha 2 chain, COMP, cartilage oligomeric matrix protein, COPII, coat protein complex II, DDR2, discoidin domain-containing receptor 2, ER, endoplasmic reticulum, HSP47, heat shock protein 47, PDB, protein data bank, PEDF, pigment epithelium-derived factor, SPARC, secreted protein acidic and rich in cysteine (also known as BM-40 or osteonectin), TANGO1, transport and Golgi organization 1

## Abstract

Collagens play important roles in development and homeostasis in most higher organisms. In order to function, collagens require the specific chaperone HSP47 for proper folding and secretion. HSP47 is known to bind to the collagen triple helix, but the exact positions and numbers of binding sites are not clear. Here, we employed a collagen II peptide library to characterize high-affinity binding sites for HSP47. We show that many previously predicted binding sites have very low affinities due to the presence of a negatively charged amino acid in the binding motif. In contrast, large hydrophobic amino acids such as phenylalanine at certain positions in the collagen sequence increase binding strength. For further characterization, we determined two crystal structures of HSP47 bound to peptides containing phenylalanine or leucine. These structures deviate significantly from previously published ones in which different collagen sequences were used. They reveal local conformational rearrangements of HSP47 at the binding site to accommodate the large hydrophobic side chain from the middle strand of the collagen triple helix and, most surprisingly, possess an altered binding stoichiometry in the form of a 1:1 complex. This altered stoichiometry is explained by steric collisions with the second HSP47 molecule present in all structures determined thus far caused by the newly introduced large hydrophobic residue placed on the trailing strand. This exemplifies the importance of considering all three sites of homotrimeric collagen as independent interaction surfaces and may provide insight into the formation of higher oligomeric complexes at promiscuous collagen-binding sites.

One of the most abundant components of the extracellular matrix is collagen (1). Until now, 28 different types of collagen have been described, which have diverse supramolecular structures and biological function in evolution and homeostasis (2, 3). Despite their functional diversity, all collagens share the presence of a defining collagenous domain, which is composed of tandemly repeated triplets Gly-Xaa-Yaa in which the Xaa and Yaa positions are predominantly occupied by proline and hydroxyproline, respectively. Proline hydroxylation is one of many posttranslational modifications that occur after cotranslational translocation of the procollagen α-chains into the lumen of the ER (endoplasmic reticulum); other examples include lysine hydroxylation and subsequent sugar attachment (4). Proline hydroxylation fosters and stabilizes formation of the collagen triple helix, where three collagen α chains first form left-handed polyproline type II helices and subsequently assemble *via* their (frequently C-terminal) prodomains into a right-handed trimeric super-helix, which is called the collagen triple helix. Here, every third residue of the α-chains is directed toward the center of the triple helix, thus resulting in the necessity for the smallest amino acid, glycine, at every third position in the primary structure. The triple helix features a stagger of the three chains resulting in a trailing, middle, and leading strand (5).

Proper triple-helix formation as well as exit from the ER of procollagen molecules depends critically on HSP47, an ER-resident chaperone belonging to the serpin family. Ablation of HSP47 leads to early embryonic death in mice (6), and several missense mutations in humans and dogs give rise to *Osteogenesis imperfecta* (7, 8, 9, 10). The exact mechanism by which HSP47 chaperones collagen folding and secretion is not entirely clear, yet. Discussed are triple-helix stabilization, prevention of lateral aggregation, as well as interaction with TANGO1, a transmembrane protein at the ER exit sites important for packaging large cargo into COPII mega-vesicles (11, 12).

HSP47 binds to collagens of different types with reported affinities in the range of 2 to 1000 nM depending on the particular collagen (13, 14, 15). The interaction involves Gly-Xxx-Arg triplets exclusively on triple-helical procollagen. The arginine residue is located in the so-called Y_0_ position and forms a crucial salt bridge with an aspartic acid of HSP47 (16). While homotrimeric procollagen molecules, therefore, always expose three potential binding sites per such a triplet, so far invariably a 2:1 HSP47:triple-helix stoichiometry has been found. In these complexes, the two HSP47 molecules bind to the leading and trailing strand of the triple helix, while the binding site of the middle strand remains unoccupied due to steric reasons (16). Negative stain EM (electron microscopy) revealed about 15 sites on the collagen I triple helix, which are distributed over the entire length, although the exact location is unknown due to the low resolution. The sites with higher affinities appear to be located toward the N-terminus of the procollagen molecules (15), and there appear some 8 to 10 HSP47 molecules bound at a concentration of 10 nM. The affinities of the individual sites will certainly depend on the amino acids neighboring the arginine residue. We will refer to individual residues according to their placement at the X and Y positions as well as to the triplet location: the arginine residue forming the salt bridge will be denoted as R_0_ and the whole binding site as G_−1_X_−1_Y_−1_G_0_X_0_**R**_**0**_G_+1_X_+1_Y_+1_. Previous studies have revealed the importance of the preceding Y_−1_ position, where, for example, a threonine residue appears favorable, while a glutamic acid abolishes binding (17, 18).

However, there is still a lack of knowledge about the influence of the amino acids downstream at the X_+__1_ and Y_+__1_ position in relation to the crucial Y_0_ arginine residue. Consequently, there is very little information on the precise location and relative affinities of the binding sites for HSP47 on the procollagen molecules.

To answer these questions, we have established a partial peptide library of the homotrimeric collagen II to further inquire the binding motifs. This library led to two new “high-affinity” binding sequence motifs, which feature a phenylalanine or a leucine at the X_+1_ position. We cocrystallized HSP47 with synthetic, homotrimeric collagen model peptides (CMPs) containing these new sites. In stark contrast to the 2:1 HSP47:triple-helix stoichiometry that has always been observed in past studies, these newly obtained crystal structures show only one HSP47 molecule bound to the site located on the trailing strand of the triple helix, while leading and middle strand positions remain unoccupied. This can be explained by the stagger of the collagen helix that leads to clashes with the second potential binding site on the leading strand.

## Results

### HSP47 binds only to five sites in the N-terminal triple-helical region of collagen II

**Figure 1:**
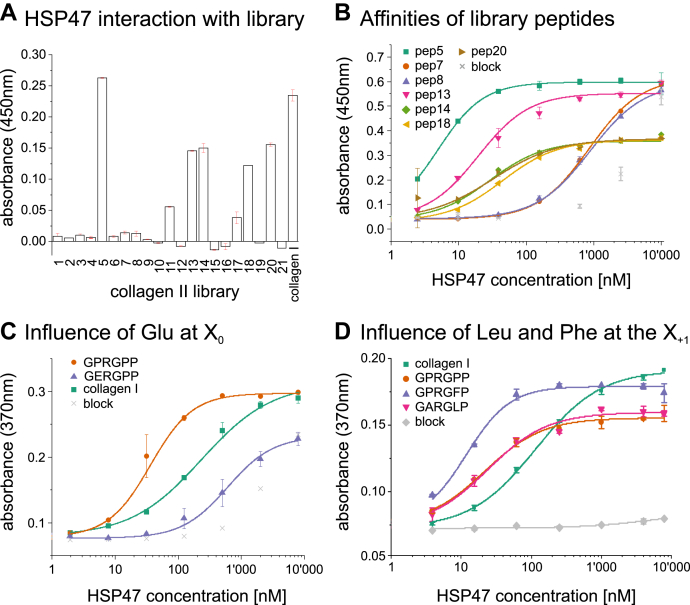
**HSP47 binds only very few peptides in the N-terminal collagen II peptide library.***A* and *B*, binding of HSP47 was investigated using ELISA-style binding assays immobilizing different collagen model peptides and incubating with soluble HSP47. *K*_D_ values for all measured library peptides can be found in Table 1. *C*, the role of a glutamate residue at the X_0_ position was investigated. *K*_D_ collagen I: 234 ± 34; GPRGPP: 36 ± 3; GER: 606 ± 322. *D*, leucine or phenylalanine at the X + 1 position does not prohibit HSP47 binding. The *K*_D_ determined b curve fittings are 21.7 ± 3, 12.0 ± 1.4, 24.1 ± 5.4 and 115.6 ± 7.6 nM for RGP, RGF, RGL, and collagen I, respectively. All measurements were performed in triplicates. In the data termed “Block,” no CMPs were immobilized, *i.e.*, they indicate the interaction of HSP47 with just the blocking reagent.

HSP47 is known to bind to arginine residues at the Y-Position of the G-X-Y triplets. However, although some studies have investigated the influence of adjacent residues on the HSP47 interaction (17, 18), none has checked the actual binding site of HSP47 on a native collagen triple helix sequence. In this study we used our established collagen II peptide library (19) spanning the first 1/3 of the collagen type II triple helix to investigate collagen binding. Here, 16 of the 20 peptides contain at least one arginine at the Y position of the collagen triplets and thus could be an interaction partner for HSP47. However, only five of those 16 peptides tested positive with varying affinities in ELISA-style binding assays (Fig. 1, *A* and *B*).

### HSP47-binding sites carry a hydrophobic residue at Y_+1_

The binding motif (G_−1_X_−1_Y_−1_G_0_X_0_R_0_G_+1_X_+1_Y_+1_) has been previously studied with respect to the position Y_−1_ using chemically synthesized peptides (17, 18). Based on these publications, ten peptides of our library (*i.e.*, pep5, pep8, pep10, pep11, pep13, pep14, pep17, pep18, pep19, and pep20) should contain at least one high-affinity site (IC_50_ < 1 μM), and two peptides (pep16, pep7) a medium-affinity site (1 ≤ IC_50_ < 10 μM). In contrast to these previous studies, we do not measure IC_50_ values, but dissociation constants (*K*_D_). These values are proportional and are related by the Cheng–Prusoff equation (20), with the *K*_D_ values always being smaller than the corresponding IC_50_ values. This explains why our high-affinity binder (GPP)_7_GPR(GPP)_7_ has a *K*_D_ value of around 30 nM, although its IC_50_ value was reported to be around 940 nM (13). In contradiction to the previously made predictions, we could only detect binding of HSP47 with good affinity to five peptides in our peptide library: pep5, pep13, pep14, pep18, and pep20 (Fig. 1*B* and Table 1). For two further peptides (pep7 and pep8) we could only detect a weak binding with an apparent affinity only slightly above the unspecific interaction of HSP47 with the blocking reagent BSA at high concentrations (Fig. 1*B*, compare with gray crosses). In a first *in-silico* approach to explain the discrepancy between the published prediction and our observation on the number of sites, we used the MutaBind2 algorithm to predict the ΔΔ*G* values for all binding sites individually based on our previously solved crystal structure of HSP47 with a GPR containing collagen model peptide (21). Some of our library peptides contain more than one potential binding site and thus have multiple predicted ΔΔ*G* values (Table 1). The collagen sequence of peptide 5, for example, is (GPP)_6_GARGFPGTPGLPGVKGHRGYPGLDGAK(GPP)_6_, which contains two potential binding sites marked by underlines. In this particular example, the first GPP triplet from the first binding site (*GPP*GARGFP) is derived from the last GPP of the host sequence and is not present in the natural collagen type II sequence. To indicate this, we marked these host-derived sequences in italics in Table 1. To our surprise, MutaBind2 predicted all collagen peptide sequences to be unfavorable for binding (ΔΔ*G* > 1.5 kcal/mol), with the exception of the motif in peptide 14, which resembles our previously cocrystallized model peptide. To understand this discrepancy, we closely compared the sequences around the central arginine (Table 1). Amino acids known to influence the affinity negatively are depicted in black (prohibiting binding), red (low affinity), and light-red (medium affinity). It is striking that many peptides, which do not bind, contain a glutamate residue at position X_0_. From a structural perspective it would be conceivable that a glutamate at this position may undergo an intramolecular interaction with the adjacent crucial arginine at position Y_0_ (potentially from a neighboring strand) and thus prevents or weakens the interaction with HSP47. However in prior studies, GER containing peptides could be pulled down by HSP47 (22). To investigate this apparent contradiction, we produced and purified a GER containing collagen peptide and measured its interaction with HSP47. As predicted from the crystal structure, there was a significantly weaker interaction with HSP47 with a *K*_D_ of 606 nM for a GER peptide in comparison to 36 nM of a GPR peptide (Fig. 1*C*). This weak recognition, however, explains why these peptides still could be pulled down in earlier studies.

**Table 1:** Arginine R_0_ containing peptides with predicted and measured affinity

^a^Possible binding motifs (GxxGxRGxx) found in respective library peptide; bold sequences indicate prediction of high or medium affinity (IC50 < 10 μM) according to previous studies (17), italics in the binding motif indicate residues derived from the host-sequences of the vector. These do not occur naturally in the collagen II sequence. ^+^ΔΔ*G* was calculated using the MutaBind2 algorithm on HSP47:CMP structure (21). Light-red, red, and black mark amino acids reported to have medium (1 ≤ IC50 < 10 μM), low (10 ≤ C50 < 100 μM), or no affinity in a GPXGPR context (17). Gray-shaded amino acids are amino acids suspected to prevent HSP47 binding. Blue-labeled amino acids are suspected to prevent binding (compare Fig. 1*D*). Green/light-green binding motifs are candidates for explaining the interaction to the related library peptide. For some peptides (*e.g.*, pep10), which do not bind, possible amino acids disturbing the binding are marked in italics. For these peptides, *K*_D_ values could not be detected. Positions without changes to the collagen consensus sequence at the G_n_ and X_n_+Y_n_ positions are indicated by empty ciricles.

Abbreviation: n.a., not applicable.

**Figure 2:**
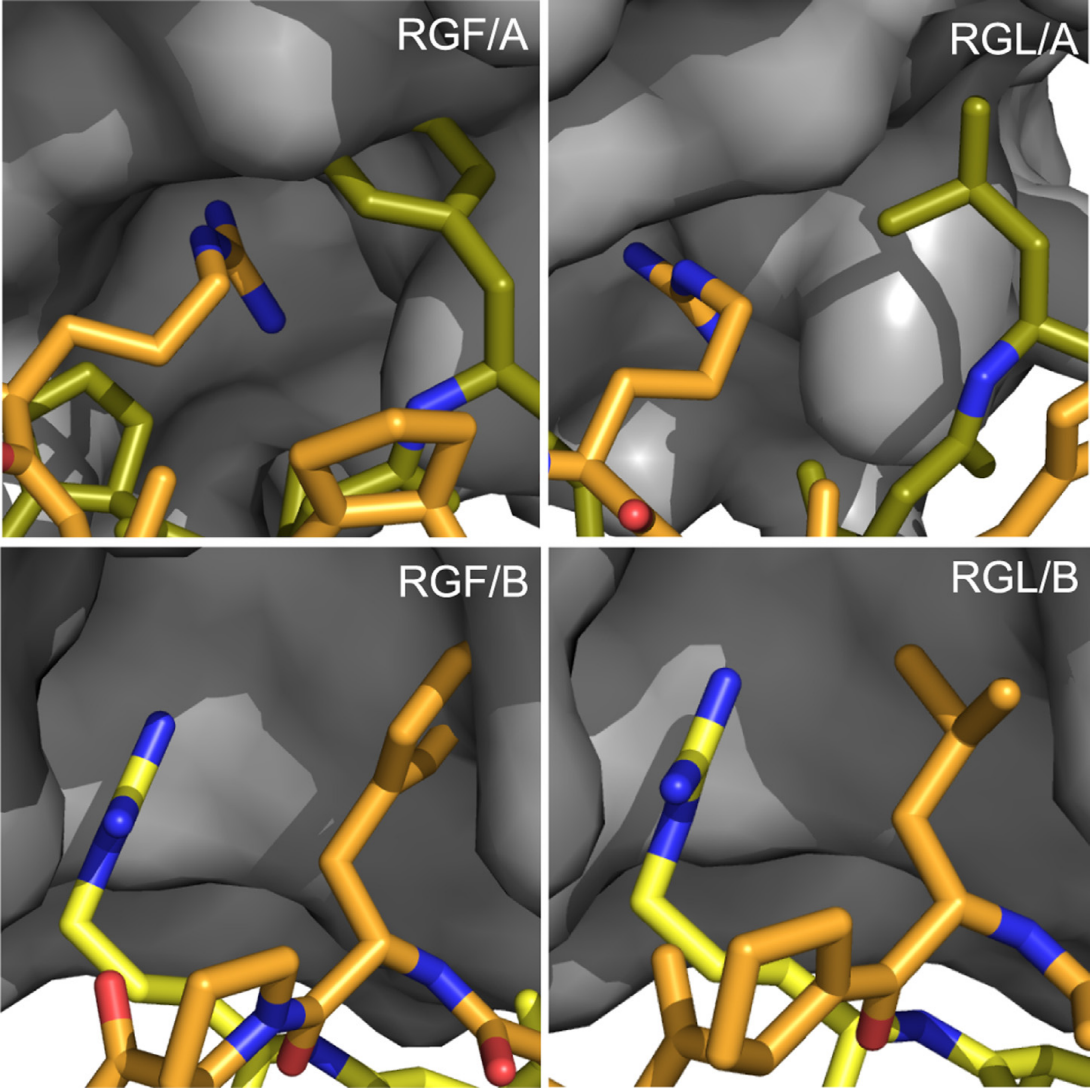
**Prediction RGF & RGL containing collagens bound to HSP47.** Amino acids at X_+1_ position were replaced by a phenylalanine or leucine respectively in our previously solved crystal structure of HSP47 and the collagen model peptide. Close-ups of the binding interface of the A- and B-site indicate clashes, which are particularly clear for the RGF peptide. The leading, middle, and trailing strands of the collagen helix are depicted in *yellow*, *green*, and *orange*, respectively.

As GER containing collagen peptides only mediate low-affinity binding, we could exclude the GER containing motifs from our analysis. This enabled us to establish a single possible binding site for most peptides from our collagen II library (Table 1 highlighted in green). Most of them are relatively rich in proline residues, but interestingly three out of five contain a hydrophobic amino acid (phenylalanine or leucine) at position X_+1_. We first investigated the effect of these hydrophobic residues *in-silico* employing the Mutabind2 algorithm (21) and the deposited HSP47 coordinates (PDB entry 4AU2). Curiously, a calculation of the effect of phenylalanine or leucine at the X_+1_ position on the binding affinity predicted an adverse effect for binding with an average ΔΔ*G* of +2.4 ± 0.6 kcal/mol for RGF and +2.9 ± 0.9 kcal/mol for RGL. Consequently, *in silico* replacement of the respective prolines in the crystal structures by phenylalanine or leucine showed clashes between these residues at the X_+1_ position and HSP47 (Fig. 2).

In stark contrast to these theoretical considerations, however, peptides containing a leucine (GARGLP) or a phenylalanine (GPRGFP) at the X_+1_ position showed in our ELISA assays similar or even slightly increased affinities compared with the GPRGPP peptide (Fig. 1*D*). To explain this observation, we determined cocrystal structures of HSP47 with synthetic model peptides containing the GPRGFP and GPRGLP motifs (from here on called RG**F** and RG**L**, respectively).

### Crystal structures of the RGF and RGL complexes show a conformational rearrangement of HSP47 forming a new hydrophobic pocket and a 1:1 stoichiometry

The complexes of HSP47:CMP[RGF/RGL] crystallized in space groups P3_2_21 and I222, respectively, which both are different from those of all previously determined HSP47 crystal structures. The structures were refined at resolutions of 1.94 Å and 2.5 Å with refinement statistics of R/R_free_ 20.3/24.8% and 21.3/26.7% (Table 2). The electron densities of the binding sites were clearly defined, and all of the important side chain orientations could be unambiguously determined (Fig. S1).

**Table 2:** Data collection and refinement statistics

Attributes	HSP47/RGF	HSP47/RGL	HSP47 HH273:274NN
Data collection			
Beamline	X06DA (PXIII) SLS	X06DA (PXIII) SLS	ID23 ESRF
Wavelength (Å)	1.00004	1.00004	0.87290
Space group	P3_2_21 (No. 154)	I222 (No. 23)	P2_1_ (No. 4)
Cell dimensions			
a, *b*, *c* (Å)	96.7, 96.7, 187.5	91.8, 129.8, 173.4	78.7, 77.4, 122.3
α, β, γ (°)	90.0, 90.0, 120.0	90.0, 90.0, 90.0	90.0, 96.6, 90.0
Resolution (Å)^a^	42.96–1.94 (2.01–1.94)	45.91–2.5 (2.59–2.5)	78.13–2.40 (2.49–2.40)
*Rmerge*^a^	0.050 (0.863)	0.12 (0.8)	0.064 (0.898)
*Rmeas*^a^	0.054 (0.94)	0.125 (0.895)	0.083 (1.16)
*CC*_*1/2*_^a^	1 (0.674)	0.997 (0.666)	0.998 (0.485)
*I*/σ*I*^a^	20.17 (1.76)	10.12 (1.57)	11.3 (1.1)
Completeness (%)^a^	99.75 (98.03)	99.02 (95.00)	97.1 (94.8)
Multiplicity^a^	6.6 (6.2)	4.4 (4.7)	2.3 (2.2)
Refinement			
Resolution (Å)	42.96–1.94	45.91–2.5	78.13–2.40
No. reflections (test set)	75,698 (7346)	36,065 (3406)	55,660 (2215)
*R*_work/_*R*_free_ (%)	20.9/24.9	21.8/26.8	23.6/26.5
No. atoms	7049	6726	12,326
Protein	6670	6647	12,182
Ligand/ion	18	6	18
Water	361	73	126
Ligands			
Protein residues	873	870	1573
Wilson-B	39.0	50.7	68.5
B-factors	43.5	54.8	63.0
Protein	43.5	54.9	68.6
Ligand/ion	50.9	53.2	80.0
Water	43.3	46.8	54.5
R.M.S. deviations			
Bond lengths (Å)	0.009	0.004	0.004
Bond angles (°)	1.45	0.96	0.77
Ramachandran			
Favored (%)	97.64	95.74	97.60
Allowed (%)	2.24	4.14	2.27
Outliers (%)	0.12	0.12	0.13
Rotamer outliers (%)	0.14	0.0	2.35
Clashscore	4	7	4
Molprobity percentile	95th	99th	100th
PDB code	7BEE	7BDU	7BFI

a Highest resolution shell is shown in parenthesis.

**Figure 3:**
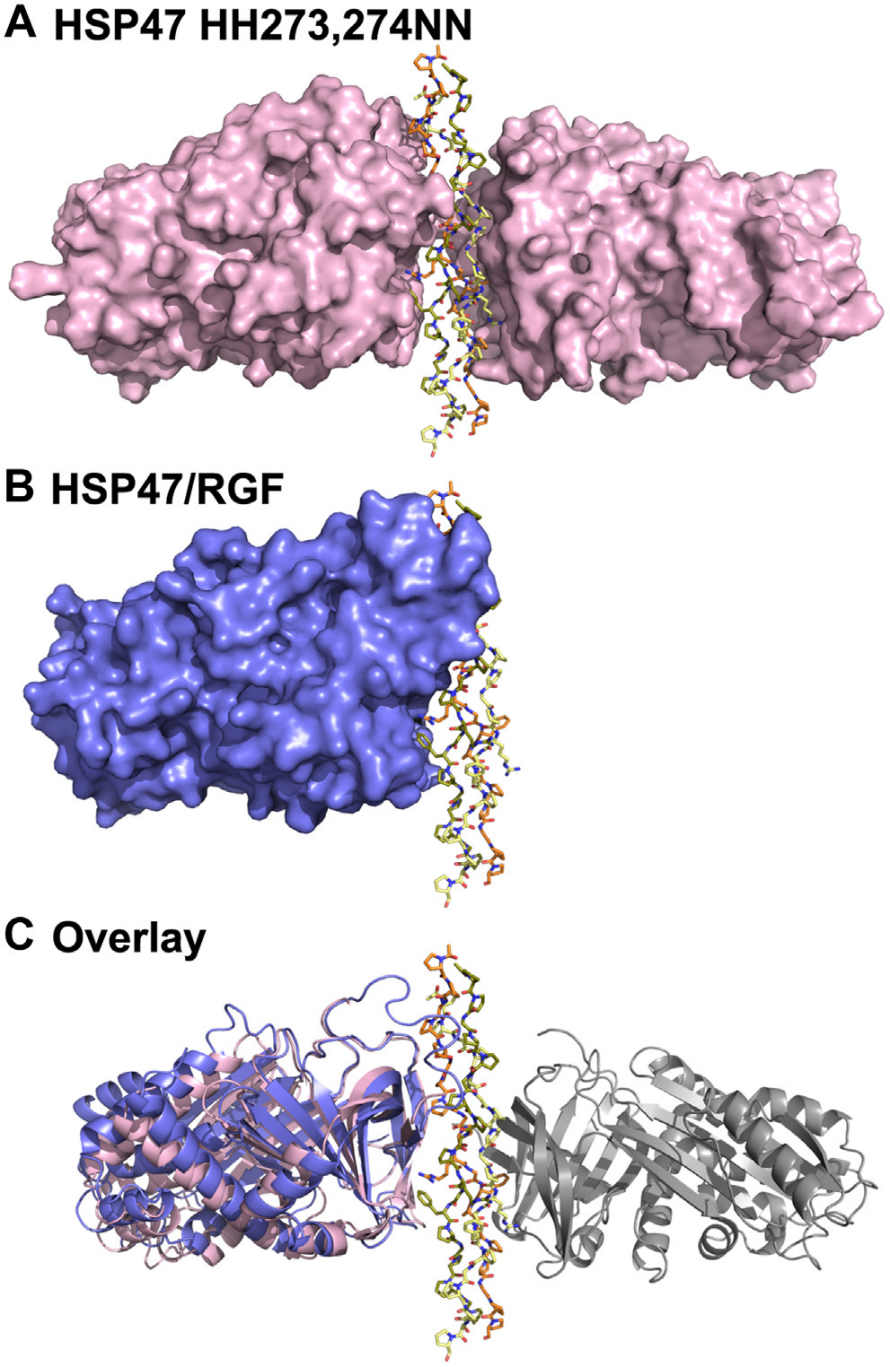
**Stoichiometry in HSP47 crystal structures.***A*, mutant and wild-type HSP47 (here the H271N/H271N double mutant shown in *pink*) always crystallized as a 2:1 complex when combined with RGP peptides of various lengths. *B*, complexes with RGL or RGF containing collagen model peptides exclusively crystallized in a 1:1 complex stoichiometry. *C*, alignment of these structures using the collagen triple helix as a fixpoint showed an overall identical binding mode (RMSD over Cα < 0.7 Å), despite the difference in stoichiometry. In *pink color* is depicted the structure with the RGP collagen model peptide, in slate color the structures of the RGF and RGL complexes, with the hypothetical second HSP47 molecule depicted in *gray color*. The leading, middle, and trailing strands of the collagen helix are depicted in *yellow*, *green*, and *orange*, respectively.

In all our previous HSP47 cocrystal structures with homotrimeric RGP motifs, the arginine residues on the trailing and leading strand each bound to one HSP47 molecule. In this paper, we will call the former interface the A-site (as it interacts with the chain A in our 4AU2 PDB structure) and the latter the B-site. There is no space for a third HSP47 molecule on the middle strand of the collagen triple helix (16). Exactly this configuration is also seen in a hitherto unpublished structure of the HSP47 double-mutant H273N/H274N, which exhibits a decreased dissociation rate off collagen at low pH (14) (Fig. 3). Although the complex of this mutant with the collagen model peptide Ac-(PPG)_2_PTGPRG(PPG)_2_-NH_2_ crystallizes in space group P2_1_, which also has not been observed in any other HSP47 crystal form so far, its structure is virtually indistinguishable from the other structures of HSP47 in complex with various collagen RGP model peptides of different length. All of them feature a proline at the X_+1_ position (PDB entries 4AU2, 4AU3, and 3ZHA (16)).

**Figure 4:**
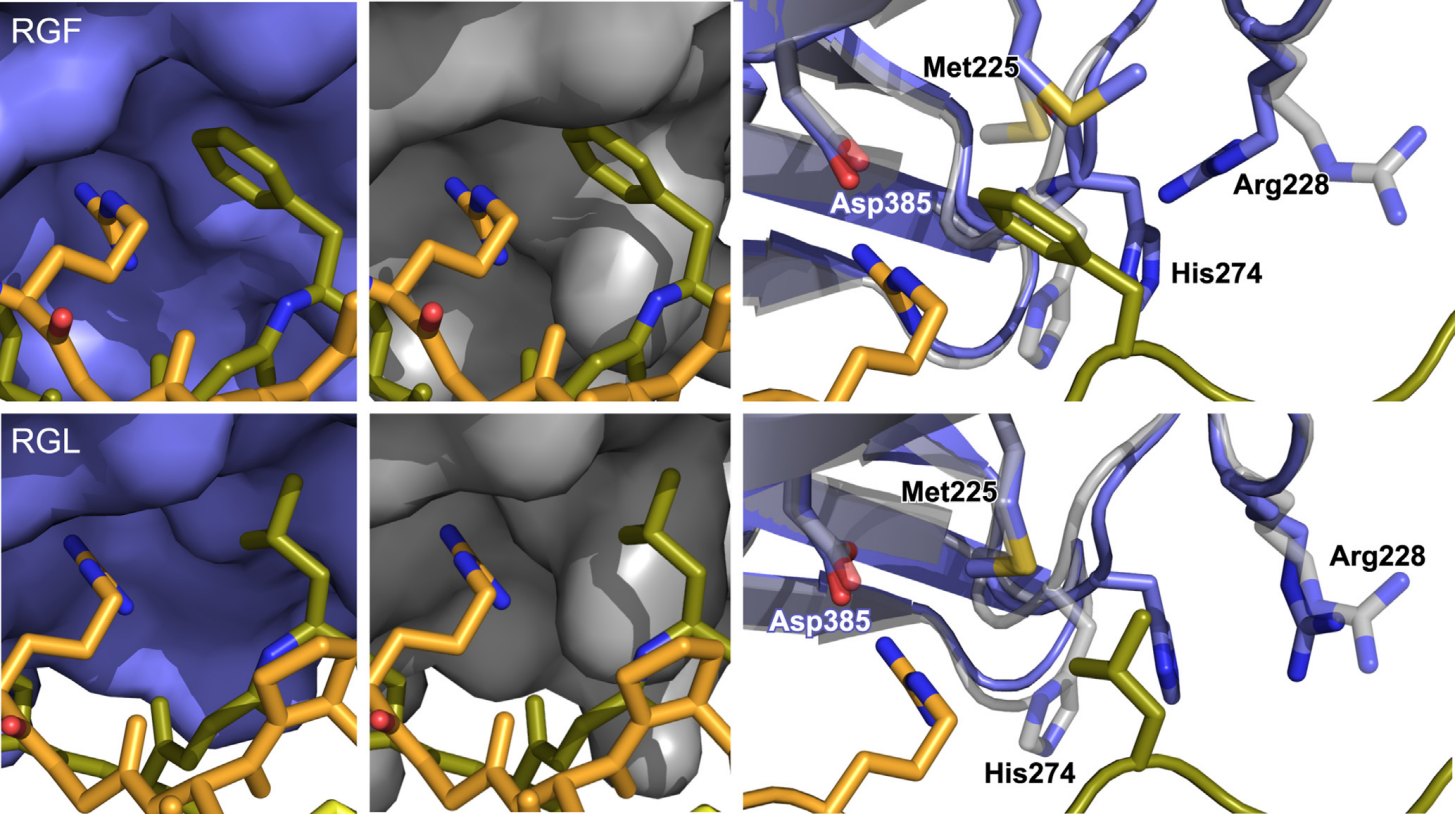
**Crystal structure of RGF and RGL containing collagen model peptides bound to HSP47.** Crystal structures of HSP47 in complex with RGF and RGL containing collagen model peptides were solved. Inspection of the interface indicated a concerted movement of the His_274_ containing loop as well as Met_225_ and Arg_228_ to create a minor groove, perfectly shielding the collagen’s phenylalanine from the solvent. The effect is less pronounced in RGL, where mostly His_274_ moves. *Left panels* show in *slate color* the RGF/RGL structures with the molecular surface. The *middle panels* indicate in *gray color* the surface of the structures of the docked RGF and RGL peptides on the previously published structures (PDB entry 4AU2). The *right panels* show an overlay of HSP47 structure of the RGF and RGL structures with the modeled HSP47 structure with an RGP peptide (PDB 4AU2). The leading, middle, and trailing strands of the collagen helix are depicted in *yellow*, *green*, and *orange*, respectively.

In contrast to all those RGP structures, these new ones employing the RGL and RGF collagen model peptides have only the A-site occupied by HSP47 (Fig. 3*B*). To adapt the bulky hydrophobic side chains at the X_+1_ position, conformational rearrangements of HSP47 take place. In both structures, the loop containing His_274_ moves slightly outward by approximately 1.8 Å (Cα – Cα distance) (Fig. 4). For the phenylalanine-containing triple helix, Met_225_ switches to a different rotamer to form a small pocket shielding the phenylalanine from the solvent (Fig. 4 upper panels). This gain of water entropy after formation of the RGF:HSP47 complex and concomitant burial of the hydrophobic side chain might also explain the slightly lower *K*_D_ observed for RGF in comparison to RGP and RGL (Fig. 1*D*).

**Figure 5:**
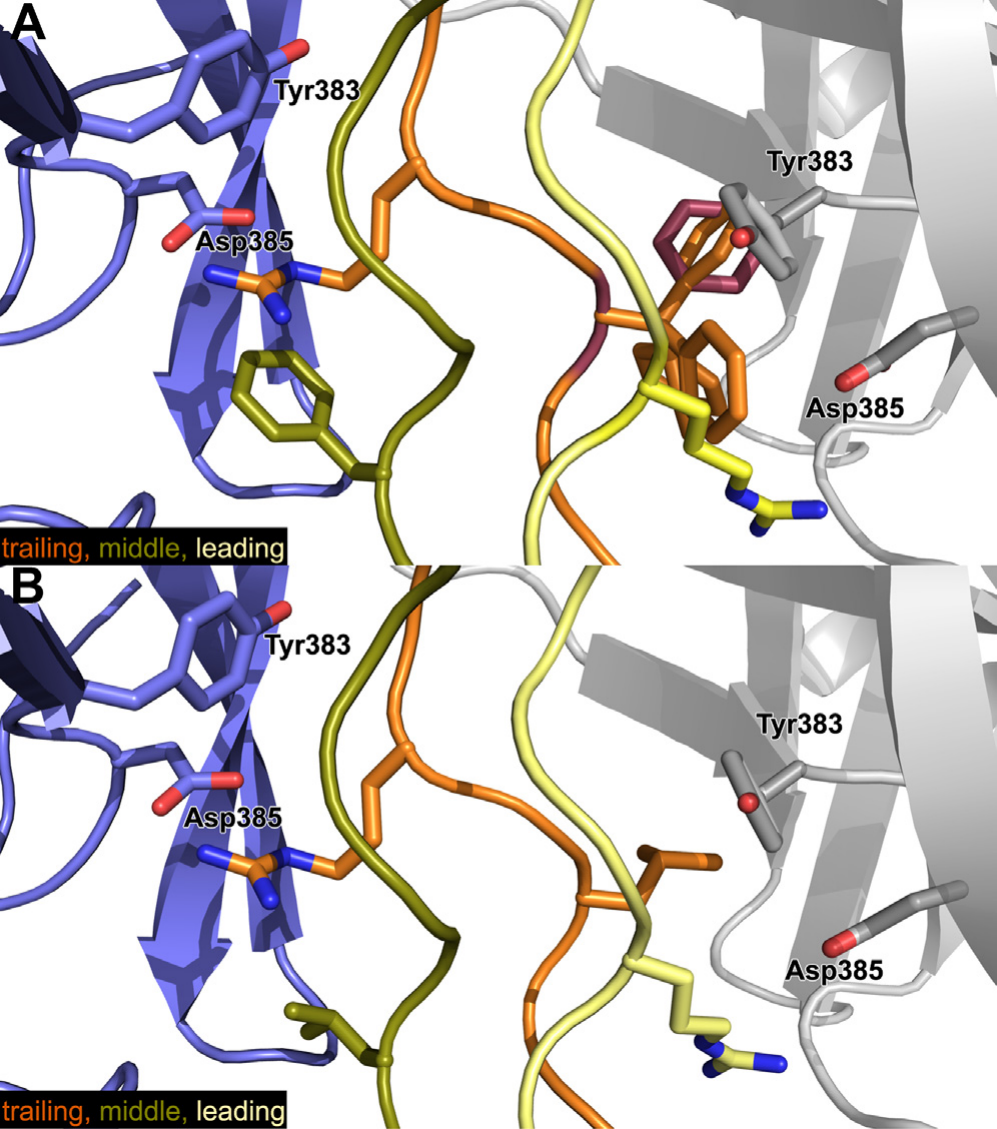
**Comparison of the 1:1 complex of the newly solved HSP47:RGF/RGL with the 2:1 complex of HSP47:RGP.** The newly solved 1:1 complexes of RGF (*A*) and RGL (*B*) were aligned to the 2:1 HSP47:RPG complex with central collagen model peptide as fixpoint. The HSP47 molecules from the B-site are colored in *gray*. To visualize potential conformations, multiple rotamers are shown for the phenylalanine in the middle strand—the rotamer found in the crystal structure is highlighted in *red* (*A*). The leading, middle, and trailing strands of the collagen helix are depicted in *yellow*, *green*, and *orange*, respectively.

Interestingly, the B-site, where in all previous crystal structures the second HSP47 molecule was bound, is empty in both of our new crystal structures, thus leading to a 1:1 stoichiometry of the collagen:HSP47 complex. To analyze if this observation is coincidental or based on the newly introduced hydrophobic amino acid at the X_+1_ position, we closely inspected the B-site of the two new complexes by docking the slightly altered HSP47 molecule from the A-sites on the respective B-positions according to the original RGP containing crystal structures (Fig. 5).

The results were similar to the prediction mentioned before (Fig. 2, RGF/B and RGL/B), the phenylalanine and—less prominently—the leucine side chain clash with either the modeled second HSP47 molecule or the collagen backbone.

The surface for HSP47 interaction on the collagen side is not formed by only a single collagen chain, but by interactions with all three strands (trailing, middle, and leading). In the RGF complex, the bound HSP47 at the A-site recognizes the arginine from the trailing chain and buries the phenylalanine from the middle strand. In contrast, on the B-site the interacting arginine would originate from the leading and the phenylalanine from the trailing strand. Owing to the staggered arrangement of the three collagen chains, this alters the spatial location of the two amino acids with respect to each other. If we orient the N-termini of our collagen helix toward the top as in Figure 5, the phenylalanine in the A-site is placed below the arginine residue (Fig. 5*A*). However, in the B-site the phenylalanine (originating from the trailing strand) is located above the arginine (Fig. 5*A*), thus the phenylalanine on the B-site contacts a different part of the HSP47’s collagen-binding interface. In that position the small cavity formed in HSP47 for the phenylalanine at the A-site is not available. Instead, the hydrophobic amino acids would clash with Tyr_383_ of the hypothetical second HSP47 protein. Additionally, in the conformation observed in our crystal structures, the phenylalanine pushes the unbound arginine out of the way so that the key salt bridge might not be able to form. A similar situation can be found for the leucine containing CMP, although the clashes appear as less severe. Still, the presence of the leucine seems to influence the positioning of the important arginine at the B-site and might prevent binding in a similar manner.

### Analytical ultracentrifugation and negative staining EM confirm a 1:1 interaction for the RGL/RGF collagen model peptides

To analyze if the newly observed 1:1 complexes are crystal artifacts or whether they also exist in solution, we examined the complex formation by analytical ultracentrifugation (AUC) and immunogold electron microscopy (EM).

For the AUC studies, foldon stabilized collagen model peptides were fluorescently labeled and incubated in various stoichiometries with HSP47. The concentration of both components always exceeded the measured *K*_D_ by at least a factor of 10.

**Figure 6:**
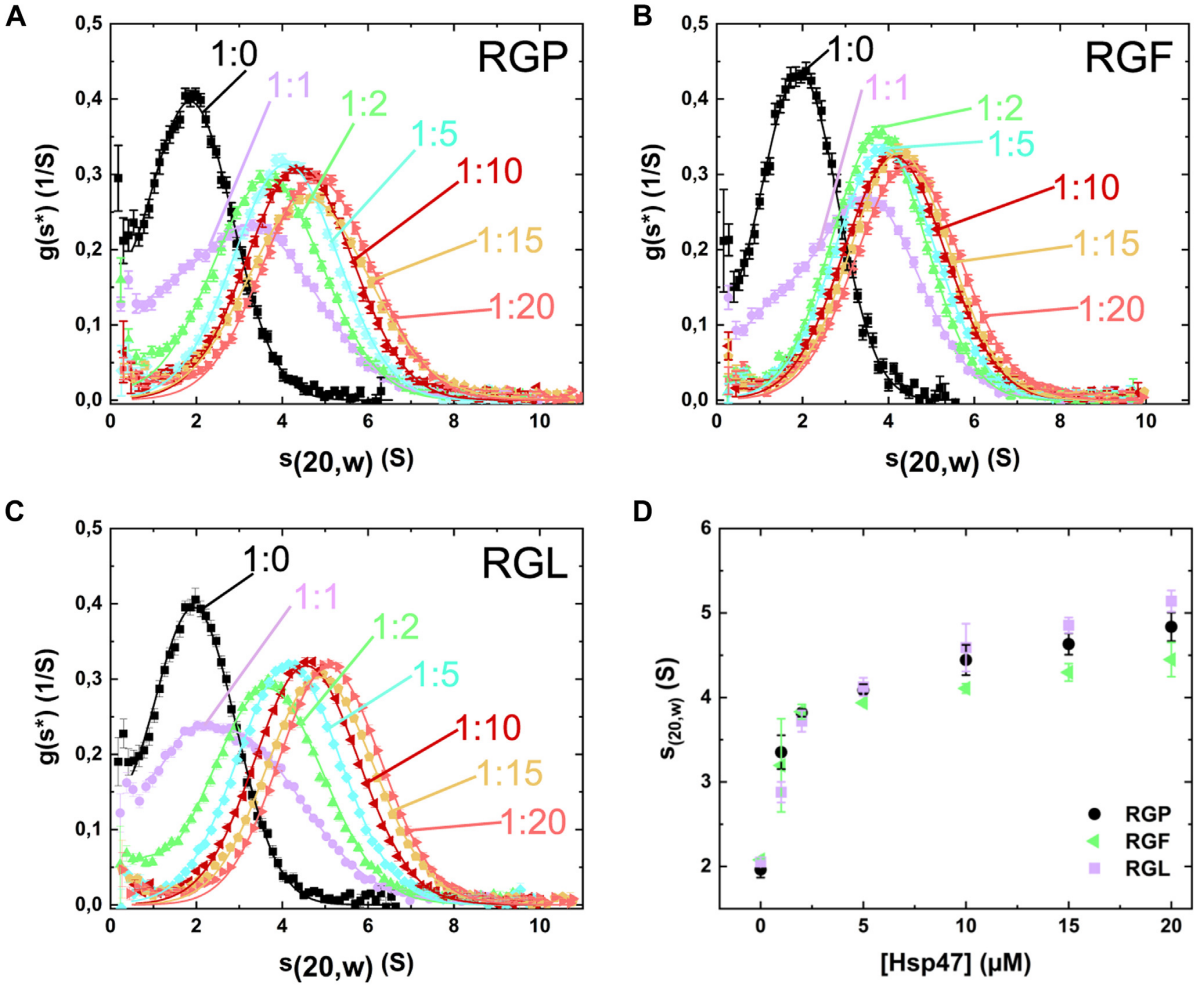
**Stoichiometry of the HSP47 complexes in solution measured by AUC.** Analytical ultracentrifugation was performed to determine the stoichiometry of the complex in solution. Collagen model peptides were fluorescently labeled and its sedimentation constant determined in dependency of the presence of different molar excess of HSP47 (*A*–*C*). For comparison, sedimentation coefficients were plotted against HSP47 concentrations (*D*). Ratios are given in CMP:HSP47; concentration of CMP was 1 μM. The g(s∗) analysis reflects the apparent sedimentation coefficient distribution of the particles in solution. For better comparability, the apparent sedimentation coefficient was normalized to water at 20 °C.

For the RGF peptide, the data from the AUC experiments are consistent with a 1:1 interaction. Similar to the wild-type at lower concentration (1–5 μM of HSP47 to 1 μM CMP), the complexes show the same sedimentation coefficient (∼4 S) as the RGP peptide (Fig. 6, *A*, *B* and *D*). However, with higher molar ratios, the sedimentation rate increased further for the RGP complex (∼5 S) indicating the formation of a 1:2 stoichiometry complex, while it stagnated for RGF. Interestingly, complexes formed with RGL behaved differently as they reached even higher sedimentation rates at high concentrations in comparison to the RGP complexes (Fig. 6, *A*, *C* and *D*).

While AUC is a good method for detecting complex sizes, we had to use rather high molar concentrations (up to 20 μM) to get a good signal. At this high concentration proteins can form unspecific interactions, a property which in our experience is particularly true for HSP47. It is also known that HSP47 weakly associates with the collagen triple helix even when there is no arginine residue present at any Y position (13, 22, 23, 24).

**Figure 7:**
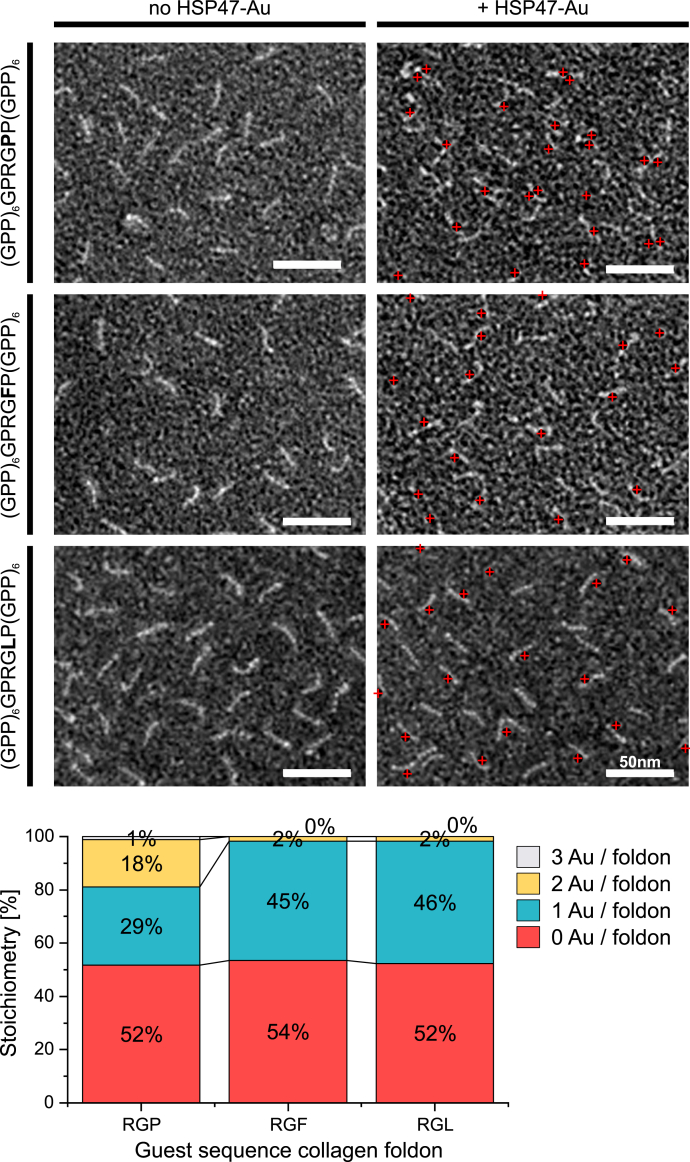
**Stoichiometry of the HSP47 complexes in solution measured by negative staining EM.** HSP47 was mixed with RGP, RGF, and RGL containing collagen model peptides and subjected to negative staining electron microscopy. For visualization HSP47 was directly coupled to 5 nm colloidal gold particles prior the experiment. For each visible collagen helix, the number of attached gold particles was counted. The EM images without markings are shown in Fig S2. Scale bars represent 50 nm.

To verify our analysis with a different method and circumvent the necessity of high protein concentrations, we also analyzed our complexes by immune-gold labeled negative stain electron microscopy. Foldon-stabilized collagen model peptides containing an RGP, RGF, or RGL motif were mixed in a 1:2 ratio with gold-labeled HSP47. Owing to the negative staining the collagen helix was clearly visible (Fig. 7). For the RGP peptide, 50% are not bound to HSP47, 29% have one, and 18% have two HSP47 molecules bound. The high amount of unbound protein is in agreement with the measured *K*_D_ for the RGP peptides of about 21 nM. For the RGL and RGF containing collagen peptides, the total amount of unoccupied foldon-CMP stays the same (52% and 54%); however, the remaining collagen helices are nearly exclusively labeled with only a single HSP47 protein (46% and 45%). These findings mirror the results of the crystal structures and demonstrate clearly that—at least at low concentrations of about 100 nM—a 1:1 complex is highly favored for RGF and RGL containing collagen peptides.

## Discussion

The exact number of HSP47 binding sites on the various types of collagens is important for a deeper understanding of the function of HSP47. In this study we have refined the HSP47 binding motifs and have shown that glutamate at the X_0_ position strongly reduces the affinity and thereby decreases the number of potential binding sites of HSP47 on procollagen molecules. For COL2A2, 24 high or medium binding sites ([TSPVA]GXRG) were predicted (17). However, after excluding sites bearing a GER sequence, only 15 of those are left.

While relatively easy for homotrimeric collagens, interpreting the effects of GER motifs in heterotrimeric collagen is more difficult if only one or two of the three sequences contain a GER motif. To estimate the effect of glutamates at the X_0_ position of a single strand, we used the MutaBind2 algorithm again to predict its effect on the HSP47 binding for all three combinations (*i.e.*, GER motif in leading, middle, and trailing strand) (21). As HSP47 can possibly bind to two sites of a collagen model peptide, we predicted the affinities of both sites (A- and B-site) independently. On the A-site, only a glutamate on the middle chain would be deleterious for HSP47 binding (ΔΔ*G* of 0.43, 2.79 and 0.34 kcal/mol for leading, middle, and trailing strand, respectively), while for the B-site a glutamic acid on the leading and trailing strands is predicted to reduce the affinity (ΔΔ*G* of 1.26, 0.24 and 1.17 kcal/mol for leading, middle, and trailing), although the values are slightly under the threshold for “deleterious” effects (>1.5 kcal/mol). This calculation can be applied to collagen type I a heterotrimer formed by two α1 and one α2 chain. The stagger was only recently reported as α1α1α2 (25). Thus, GER triplets at the α1 chain (*i.e.*, glutamates at X_0_ position in trailing and middle strand) would negatively influence both binding sites and potentially recreate our finding for the reduced affinity of GER motifs in homotrimeric collagen (Fig. 1*C*), while GER triplets only in the α2 chain (*i.e.*, glutamates only at X_0_ position in the leading chain) might only affect the affinity toward the B-site. The resulting collagen helix should still be able to bind at least a single HSP47 molecule at the A-site, thus forming a 1:1 complex. In total, we find 34 positions on the collagen I helix where there is a high-affinity binding site ([TSPVA]GXRG) in at least one of the chains. Three out of those have a GER motif on both chains, seven have a GER only on the α1 chain, and two have a GER motif on the α2 chain. However, both of the latter have a negatively charged amino acid at the respective position at the α1 chain, which could have a similar negative impact on HSP47 binding as a GER triplet. Incorporating these findings, it is feasible to estimate about 16 to 22 binding sites for HSP47 on collagen type I. This also fits better with the approximately 15 observed HSP47 molecules decorating a collagen I helix as observed in a previous study (15). Similar calculations could be performed for all other known collagen clients of HSP47 (15).

Although not formally described until now, the conclusion that heterotrimeric collagens might control the stoichiometry of complexes by their primary structure does not come as a surprise. However, similar effects were not known for homotrimeric collagens prior to our study.

### Homotrimeric collagen can modulate the complex stoichiometry by its amino acid sequence

Collagen triple helical domains form a staggered super-helix. It is common knowledge that even homotrimers form three independent interfaces with different topology for interaction partners (5, 26). However, to our knowledge this is the first time that a collagen binder interacts with slightly different sequences not only with different affinity but also with different stoichiometry. So far, HSP47 has always been observed to form a 2:1 complex with homotrimeric collagen model peptides of the sequence (PPG)_n_PRG(PPG)_n_. To accommodate a phenylalanine or a leucine residue at the X_+1_ position, several amino acids on HSP47 have to slightly shift their position to make additional space. However, while on the A-site HSP47 is able to make the necessary adjustments, especially with the movement of the loop containing His_274_, on the B-site the newly introduced hydrophobic residues collide with completely different amino acids of HSP47 (mainly Tyr_383_). Obviously, HSP47 lacks the flexibility in that part of the protein to adjust to the altered ligand. It is currently unknown whether other amino acids (such as isoleucine) might have similar effects on the complex stoichiometry. As a prerequisite the amino acids need to be small enough to fit into the new cavity formed by HSP47 at the A-site, but big enough to produce unfavorable steric hindrance at the B-site. Furthermore, the small cavity is slightly hydrophobic and thus many strong polar amino acids might not be good candidates.

RGF/L triplets are sparsely distributed over the different collagens. In COL1A1 and COL1A2, two and three out of 16 and 23 high and medium binding motifs have a leucine or phenylalanine at the X_+1_ position. Similarly, in COL2A1, three out of 15 putative HSP47-binding sites have an RGF or RGL motif. The highest content of RGF/RGL motifs can be found in COL5A2 with five out of 20 HSP47 recognition sites.

### The phenylalanine pocket

Phenylalanine is not an uncommon amino acid in collagen recognition motifs. Specifically, SPARC (secreted protein acidic and rich in cysteine) and DDR2 (discoidin domain-containing receptor 2) both undergo spatial rearrangements upon collagen binding to form a dedicated specificity pocket (called a “Phe pocket”) to shield the hydrophobic residue from the solvent (27, 28). However, for these two proteins the presence of a phenylalanine in the collagen sequence is essential for binding and the rearrangements in the proteins occur during the transition from unbound to bound protein. For HSP47, formation of the small cavity is facultative for binding and only mildly affects the affinity. Both SPARC and DDR2 are currently only known to form 1:1 complexes and do not show a sequence-dependent stoichiometry.

The I domain of integrin α2 also binds to phenylalanine containing motifs (GFOGER, where ‘O’ stands for l-4-Hydroxyprolin) and also rearranges upon binding. However, in contrast to DDR2 and SPARC, the I domain does not shield the phenylalanine from the solvent. Interestingly for the I domain, crystal structures describing a 1:1 and a 2:1 complex are reported, not dissimilar to the situation in HSP47 (28, 29). However, here the change occurs in the integrin domain. While the wild-type only forms a 1:1 complex, the activating mutant E318W also forms complexes of 2:1 stoichiometry.

Very recently, the crystal structure of the PEDF–collagen (pigment epithelium-derived factor) complex was described (30). PEDF has a much longer recognition sequence in comparison to HSP47; however, it also contains the RGF motif. It is interesting that HSP47 and PEDF are both serpins and although they have evolved a different binding mode for collagens, both do recognize an RGF motif at the core interface. It is also noteworthy that all binding sites for PEDF contain an RGF-modulated HSP47-binding site. Although PEDF is in principle an extracellular protein, both proteins will compete for the same binding site inside the ER during PEDF’s secretion pathway. Structural comparison of our newly generated HSP47 complex formed on RGF peptides with the PEDF–collagen complex reported earlier predicts that both binding sites are mutual exclusive. These particular sites on collagen type I and II are further known to also interact with COMP (cartilage oligomeric matrix protein) (19), heparin (31) and are important for cross-linking (32, 33). For COMP the intracellular binding is reported to be beneficial for collagen secretion (34). Currently, the 3D structure of the COMP:collagen complex is unknown; however, having only a single HSP47 protein bound to an otherwise promiscuous binding site might enable the formation of ternary complexes and thus facilitate secretion. Future studies will be necessary to shed light on the potential role of our newly discovered stoichiometry-controlled binding sites for collagen secretion.

## Experimental procedures

### Protein production and purification

The collagen model peptides were generated and produced as described previously (19). Shortly, peptide sequences were back-translated and cloned into pCMP-3b, a pET-based vector, designed in the aforementioned study. The final construct contained a C-terminal T4 foldon domain for trimerization (35), an N-terminal 2xStrep tag for purification and a (GPP)_6_-guest-(GPP)_5_ sequence for collagen helix formation. For the detailed studies, the guest sequence consists of GPRGPP, GARGLP, and GPRGFP respectively.

HSP47 was produced and purified as described earlier (14). Briefly, a *Canis lupus* derived, codon-optimized construct of HSP47 was cloned into the pET22-(b) vector (Novagen), encoding residues _36_LSP … RDEL_418_ with a C-terminal hexahistidine tag.

Production of all aforementioned proteins was done in the *E. coli* BL21 (DE)_3_ strain. Cells were grown to an OD600 of 0.6 to 0.7 and expression was induced with 0.5 mM isopropyl-β-d-thio-galactopyranoside for HSP47 and 1 mM for the collagen model peptides. Cells were harvested after shaking for 5 h at 37 °C (HSP47) and after overnight expression at 20 °C (collagen model peptides).

For purification, cells were resuspended in phosphate buffered saline with addition of DNAseI and AEBSF (4-(2-aminoethyl)benzenesulfonyl fluoride hydrochloride) and were lysed using a cell disruptor (CF Cell Disruptor CF1 model, Constant Systems Ltd) at 2.5 bar. After centrifugation the cleared supernatant was purified using immobilized metal affinity chromatography (HSP47, Ni-NTA superflow, Qiagen) and affinity chromatography (CMPs, Strep-Tactin System, iba). Dithiothreitol (DTT) was added to a final concentration of 4 mM to the HSP47 eluate, and contaminants were precipitated with 1.5 M ammonium sulfate. All proteins were loaded to a Superdex 200 Increase 10/300 GL (GE LifeSciences) as a last purification step and to exchange the buffer to 20 mM HEPES, 150 mM NaCl pH 7.5, and 4 mM DTT in case of HSP47. HSP47 was concentrated to ∼20 mg/ml and CMPs to 3 mg/ml using Amicon Ultra centrifugal filters (Merck) with 30 and 10 kDa weight cutoffs, respectively. All proteins were stored at −80 °C until further use. Protein concentrations were measured by absorption at 280 nM with absorption coefficients calculated based on their protein sequence, *e.g.*, 42,400 M^−1^ cm^−1^ for our HSP47 construct (*M*_r_ 44,099 Da). Proteins were checked for purity *via* SDS-PAGE and Western Blot.

### Enzyme-linked immunosorbent assay (ELISA)

Purified collagen model peptides were coated onto a Nunc MaxiSorp (Thermo Fisher Scientific), 500 ng/well. Plates were washed after 1 h at RT with TBS-T 0.05% and free binding sites were blocked with 1% (w/v) BSA in TBS. The collagen type II library was first screened with single point measurements at a HSP47 concentration of 1 μM and positive hits further analyzed by titration experiments. In all titration experiments, the analyte exposure consisted of a 1:4 dilution series of HSP47, starting at 8 or 10 μM. HSP47 was detected by anti-His-HRP (1:10,000 in 1% BSA in TBS-T 0.05%, Miltenyi Biotec) and developed using freshly prepared TMB (3,3′,5,5′-Tetramethylbenzidin) solution. Assays were either stopped with 10% H_2_SO_4_ and absorption measured at 450 nm or continuously monitored at 370 nm every 20 min for 1 h. In latter cases, timepoints showing the highest signal-to-noise ratio were used for analysis. As a positive control collagen type I from rat tail collagen (Corning) was used. Results were analyzed using Gen5 (BioTek) and Origin 2018.

### Cocrystallization of HSP47 and synthetic collagen peptides and structural modeling

HSP47 was cocrystallized with synthetic homotrimeric model peptides (ACE-PPGPPGPPGP**R**G**[F,L]**PGPPGPPGNH_2_, Peptide 2.0) in 0.1 M HEPES, 18 to 26% PEG 3′350 and 1 to 6% Tacsimate pH 7.0 (Hampton). The drop ratio varied between 2:1, 1:1 and 1:2 (protein:reservoir) with a total drop volume between 200 and 300 nl. Collagen model peptides were added in a molar ratio of 1(HSP47):3(trimeric CMP), with final concentration of 400 and 1200 μM, respectively. Peptide concentrations were determined based on the dry weight supplied by the manufacturer. The cryoprotectant contained the mother liquor mixed with glycerol added to an end concentration of 25% (vol/vol).

Data was collected at the PXIII at the SLS, Switzerland Swiss Light Source (Paul Scherrer Institute). All datasets were processed using XDS (36), and the structures were solved by Molecular Replacement using PHASER (37) implemented in the phenix package (38) with a single HSP47 molecule of PDB entry 4AU2 (16) as search model. All structures were refined using iterative cycles of phenix.refine and Coot (39). For structural analysis and figure generation, the open-source version of PyMOL (The PyMOL Molecular Graphics System, Version 2.1 Schrödinger, LLC) was used. Coordinates of complex were deposited in the PDB database under the accession number 7BDU, 7BEE, and 7BFI for the RGL, RGF, and HH273:274NN containing crystals, respectively.

Calculation of ΔΔ*G* for collagen peptides was performed with the MutaBind2 algorithm (21). The crystal structure of HSP47:CMP from our previous studies (4AU2) was used as the starting point. The asymmetric unit was reduced to one collagen molecule (chain E+F+G) and one HSP47 molecule per calculation (either chain A or chain B). Mutations were introduced in three chains of the collagen helix and the respective ΔΔ*G* for chain A and chain B of HSP47 was calculated separately. Complexes formed with chain B consistently showed worse ΔΔ*G* values; thus, only values for chain A are presented.

### Analytical ultracentrifugation

The purified collagen model peptides were labeled with ATTO488 for 1 h at 37 °C. Unbound fluorophore was removed using NAP-5 and PD-10 columns. Proteins were mixed in 20 mM HEPES, 150 mM NaCl (pH 7.5) in the following ratios (CMPs:HSP47): 0:1, 1:1, 1:2, 1:5, 1:10, 1:15, 1:20. All samples were incubated for 30 min at RT and afterward centrifuged at 42.000 rpm in a ProteomeLab XL-A analytical ultracentrifuge (Beckman Coulter) equipped with a fluorescence detection system (AU-FDS; Aviv Biomedical Inc). In this setup 42.000 rpm translates to 117.552*g* at the top (r = 59.5 mm) and 142.248*g* at the bottom (r = 72.0 mm) of the sample cell. Samples were spun in standard 12 mm double-sector epon-filled centerpieces, covered with quartz windows. All measurements were performed in triplicates and key findings repeated three times. Representative results are shown.

The resulting sedimentation velocity profiles were analyzed using DCDT+ version 2.4.3 software by John Philo (40). The buffer density (ρ) and buffer viscosity (η) of the buffer used for data analysis were calculated with SEDNTERP 20120828 BETA (41).

### Transmission electron microscopy

HSP47 was conjugated with 5 nm colloidal gold (42) and incubated with foldon-stabilized collagen model peptides in a 2:1 ratio at a concentration of 200 nM and 100 nM, respectively. The binding was visualized by negative staining and transmission electron microscopy as described earlier (43). Briefly, mixed protein solutions were adsorbed to 400 mesh carbon-coated copper grids and stained with 0.75% (w/v) uranyl formate. Specimens were examined in a Philips/FEICM 100 TWIN transmission electron microscope operated at 60 kV accelerating voltage. Images were recorded with a side-mounted Olympus Veleta camera with a resolution of 2048 × 2048 pixels (2k × 2K) and the ITEM acquisitions software. Binding frequency was determined by analyzing 30 fields of 1 μm^2^ counting all collagen model peptides with 0, 1, 2, and 3 HSP47 molecules bound. As control, also free HSP47 molecules were counted.

### Quantification and statistical analysis

The values shown for ELISA-style binding assays represent the average of triplicates. Data from affinity assays were analyzed using a four-parameter logistic fit function (44) in Origin 2018.

## Data availability

All data regarding the crystallographic structure determination are available from the PDB database under the accession numbers 7BDU, 7BEE, and 7BFI. All other data are included in this manuscript.

## Supporting information

This article contains supporting information.

## Conflict of interest

The authors declare that they have no conflicts of interest with the contents of this article.
