# Supplementary material for: Collagen’s primary structure determines collagen:HSP47 complex stoichiometry
Source: J Biol Chem. 2021 Sep 4;297(6):101169. doi: 10.1016/j.jbc.2021.101169 (PMC8626583; doi:10.1016/j.jbc.2021.101169)
Supplement: Supplemental Figures S1 and S2 [file mmc1.pdf]

Supporting information for

Collagen’s primary structure determines collagen:HSP47 complex stoichiometry

Elena T. Abraham, Sinan Oecal, Matthias Mörgelin, Philipp W. N. Schmid, Johannes Buchner, Ulrich Baumann, Jan M. Gebauer

Contents:

- Supplemental figure 1
- Supplemental figure 2

Supplemental figure 1

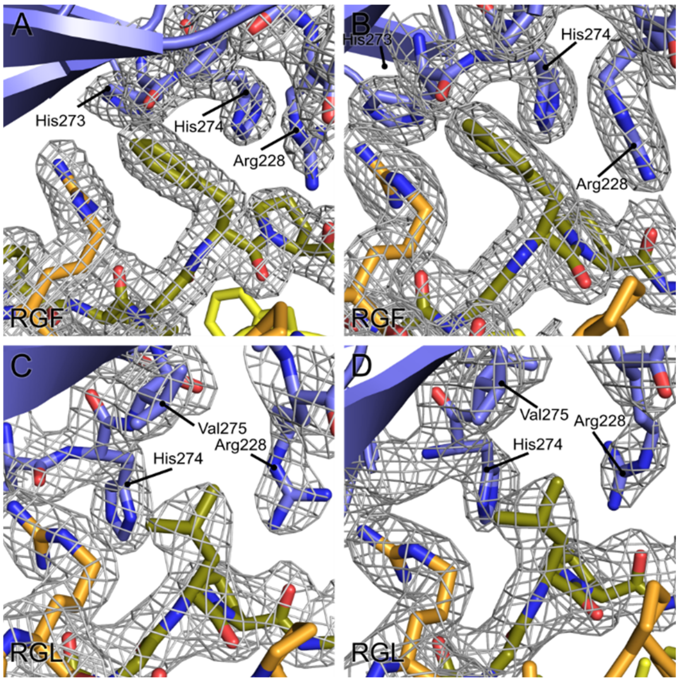

Supplementary Figure 1: Electron density maps of the most important residues in the binding interface

Electron density maps contoured at 1.0σ for the central binding interface of HSP47:RGF (A-B) and HSP47:RGL (C-D). Both crystal forms contain two protomers per asymmetric units, which a very similar (left and right panels). Collagen model peptide with the central arginine and a phenylalanine/leucine residue are shown. The leading, middle, and trailing strand of the collagen helix are depicted in yellow, green, and orange, respectively.

Supplemental figure 2

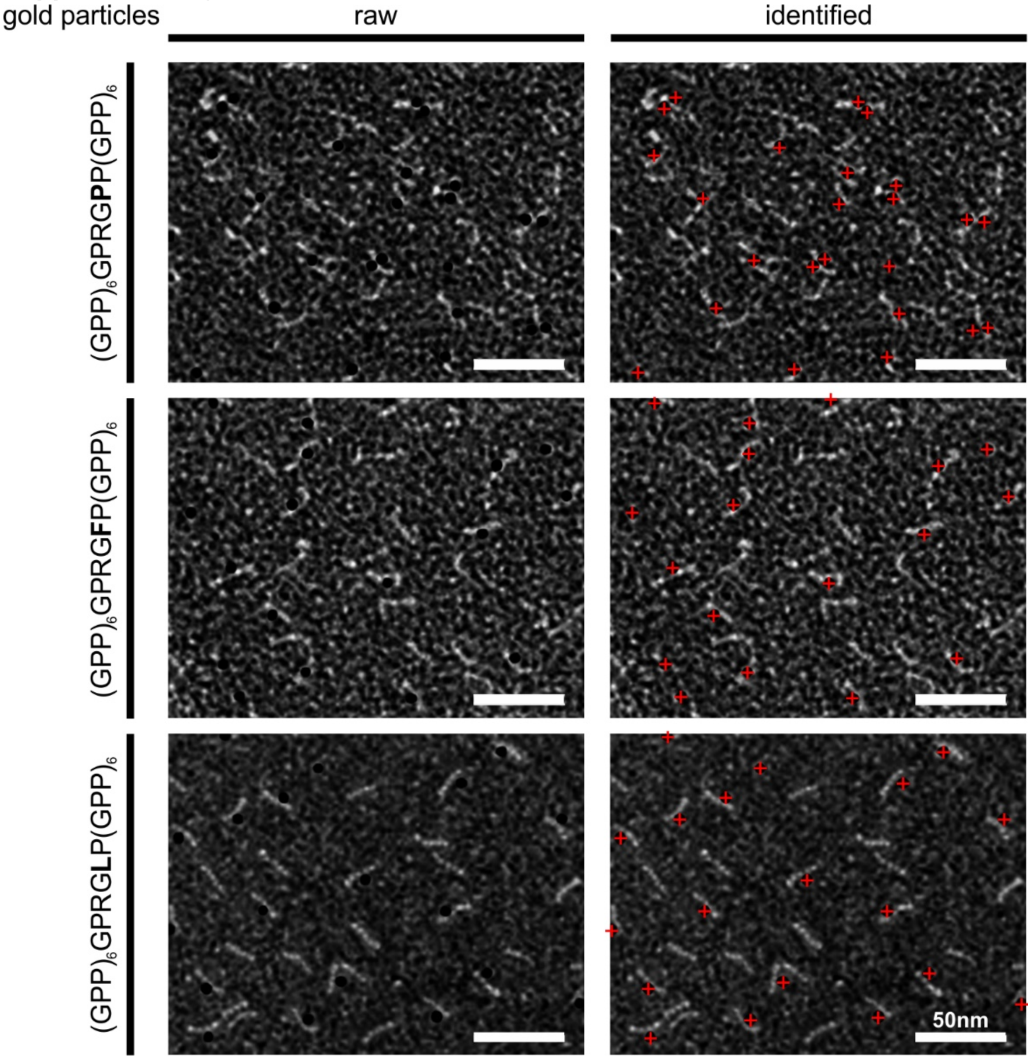

Supplementary Figure 2: Visualization of HSP47 complex formation in dependency of the sequence of the collagen model peptide

These images are identical to those shown in figure 6, with the exception that the left panel lacks the +-signs indicating the identified HSP47-Au particles. Scale bars represent 50 nm.
